# DHF: A Generative AI Approach to Data-Efficient Signal Separation for Wearable Embedded Systems

**DOI:** 10.1145/3820374

**Published:** 2026-08-24

**Authors:** MAHYA SAFFARPOUR, ZIYUAN LI, KOUROSH VALI, WEITAI QIAN, BEGUM KASAP, SOHEIL GHIASI

**Affiliations:** Electrical and Computer Engineering, University of California Davis, Davis, United States; University of California Davis, Davis, United States; University of California Davis, Davis, United States; University of California Davis, Davis, United States; University of California Davis, Davis, United States; Electrical and Computer Engineering, University of California, Davis, Davis, United States

**Keywords:** Wearable systems, signal separation, quasi-periodic signals

## Abstract

Separating sensed signals is a crucial task for many wearable systems, where the acquired data contains a mixture of a target signal of interest and several interfering signals, arising from quasi-periodic physiological phenomena such as cardiac and respiratory activity with slowly varying frequency and amplitude. In practice, the task of separating the signals is challenged by quality and quantity of available physiological data. To overcome this limitation, we propose a novel signal separation technique that can effectively isolate the target signal from a sensed mixed signal, using only a single trace of data. We leverage application-specific attributes of the problem, and approach signal separation as an instance of spectrogram in-painting with a custom-designed generative neural network model. The efficacy of the method is demonstrated in the context of transabdominal fetal pulse oximetry application, using both synthesized and *in-vivo* data collected from pregnant ovine models. Our approach significantly outperforms prior art, achieving 106% average improvement in signal-to-distortion ratio and 85% reduction in mean squared error in synthesized data, compared to the best competing method. In case of *in-vivo* data collected in animal experiments, where reference signals are unknown, our method substantially improves performance of a downstream task. Specifically, the proposed method reduces the correlation error in estimated fetal blood oxygen saturation by 80.5% compared to a state of the art technique, showcasing its potential to advance wearable systems.

## 1 Introduction

Wearable systems have emerged as a promising technology for advancing personalized and proactive healthcare by enabling continuous health monitoring, improved diagnostics, and timely interventions [27]. Wearable systems utilize sensors to obtain information about a signal of interest, which typically has some physiological significance. In practice, however, the acquired data may be made up of multiple constituting signals, which can confound the signal of interest (aka target signal).

The separation of signals, a well-explored task in domains such as audio and speech processing where abundant high-quality data is available, faces challenges in wearable applications due to the cost and regulatory barriers associated with collection of large multi-sensor physiological datasets [3]. For example, in applications such as tissue oximetry or blood glucose monitoring, interfering signals, such as those from venous pulsation, respiration, or Mayer waves, can dominate the signal of interest due to arterial pulsation. Furthermore, the collected data may face limitations in both labeling, due to unknown ground truth physiological state, and dimensionality, due to the limitation on the number of sensors that can be practically worn on the body. As a result, a growing number of wearable applications need signal separation methods that rely on fewer channels of data, and can handle small, unlabeled datasets efficiently.

Having to function with limited and under-representative data, a generalizable signal separation method must take advantage of the prior, application-specific knowledge, to fill the gap. For example, one may utilize application-inspired loss functions, or embed prior information within the neural network’s structure. Integrating statistical priors directly into a loss function, the traditional approach, implicitly relies on accurate modeling of complex real-world phenomena. On the other hand, embedding priors in the neural network’s architecture, such as the “deep image prior” method [31], provides greater flexibility as the focus will be on constraining the neural network model to meet application requirements, providing a promising avenue for maintaining generalizability under limited data.

In this article, we propose a signal separation method, named **Deep Harmonic Finesse (DHF)**, that leverages application-specific properties in time-frequency representation of the signal, to separate single-channel data streams assuming (1) signals are non-stationary and quasi-periodic, (2) only one trace of single-channel data is available, and (3) signal amplitudes are unknown, however, the fundamental frequency of each signal source is either known through auxiliary sensing, or is obtainable through a pre-processing step [13, 20, 34]. For example, if a signal is regulated by the cardiac cycle, we assume that the heart rate of the subject is already known, either through a preprocessing step, or using an auxiliary measurement.

Notably, the proposed method addresses two major challenges: (1) the potential overlap and crossover of dominant frequency contents of the signals (i.e., in-band noise), which renders classic frequency-based filtering ineffective; and (2) the limitation of having only a single trace of data, which precludes the use of traditional machine learning methods that require a sizable training dataset. We use the terms trace or sample, to refer to the data acquired on one subject in one recording data frame, which can range from several to tens of minutes in duration in typical applications.

We approach signal separation as a task of masking non-target signals in the time-frequency space, and in-painting areas of spectral overlap or intersection with the target signal. Specifically, the proposed method consists of two orchestrated components, the adaptive pattern alignment unit and the complementary generative neural network structure. To isolate a non-stationary quasi-periodic target signal from the mixed input, the pattern alignment unit adaptively resamples the mixed signal according to the changing target frequency, ensuring that the target signal will have a strictly periodic pattern in the transformed (resampled) space. Subsequently, the resampled trace is fed into a complementary generative neural network, which enables efficient separation of the target signal, using a random seed value. A separate step of the proposed method interpolates the phase data, which is used to compute an inverse transformation from the in-painted spectrogram to the time domain, and to obtain the separated target signal.

## 2 Motivating Example

Current approach to fetal monitoring relies on **fetal heart rate (FHR)** trace interpretation. This method is widely acknowledged to have a high rate of false positives for detection of fetuses at risk of hypoxic distress. As a result, there has been a rise in the rate of unnecessary interventions, such as emergency Cesarean section surgeries, without corresponding improvements in maternal and neonatal health outcomes [5, 19]. Continuous monitoring of fetal blood oxygenation, as a supplemental source of information, has the potential to address the problem with the present approach. In particular, **Transabdominal Fetal Pulse Oximetry (TFO)** is an investigational method for continuous and non-invasive measurement of fetal blood oxygen saturation. TFO may be viewed as an extension of conventional pulse oximetry, where the objective is to infer fetal arterial blood oxygen saturation from outside of the maternal abdomen [4].

**Fig. 1.**
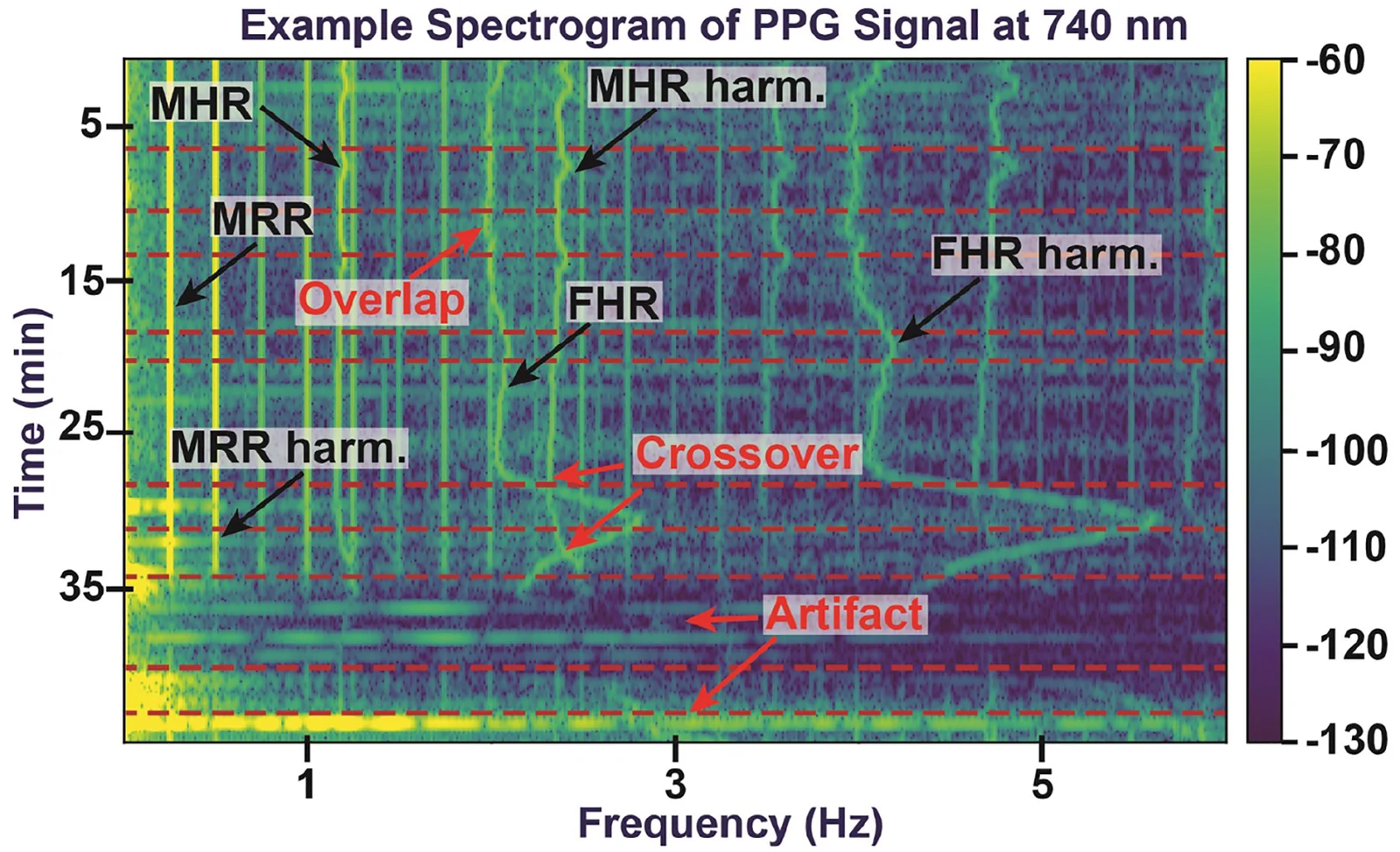
Example mixed PPG signal spectrogram obtained from a pregnant ewe model. The mixed signal is influenced by three quasi-periodic physiological phenomena: ewe’s respiration, ewe’s cardiac cycle, and fetal lamb’s cardiac cycle. The fundamental frequencies of these signals are labeled as Maternal Respiratory Rate (MRR), Maternal Heart Rate (MHR), and Fetal Heart Rate (FHR). Higher order harmonics of the signals are also visible in the spectrum.

Figure 1 illustrates an example **short-time Fourier transform (STFT)**, also known as spectrogram, of **Photoplethysmography(PPG)** data acquired in an approximately 40 minute long experiment by the TFO device. The experiment is further discussed in Section 5.3. PPG refers to the non-invasively acquired light intensity waveforms, which temporally change as a function of motions and subtle changes to the blood composition, such as hemoglobin concentration in the light path and blood oxygen saturation [26].

The figure illustrates that several physiological phenomena, such as ewe’s respiration-induced motions, ewe’s cardiac cycle, and the cardiac cycle of the fetal lamb, cause temporal changes in the acquired PPG, and are thus, mixed in the sensed data. Physiological signal tend to have complex shapes, and thus, they tend to have multiple harmonics in the signal spectrum. In particular, the strong ventilator-induced regular respiration of the ewe, at the rate of about 0.25 Hz or 15 breath per minute, has many harmonics in the illustrated spectrogram.

As a precursor to computing fetal blood oxygen saturation, fetal PPG signal needs to be separated from the acquired mixed signal, which also contains contributions from the superficial maternal tissue. The signal separation problem is exacerbated due to the low signal-to-noise (SNR) of fetal PPG signal, and its weakness relative to maternal tissue contributions to the sensed signal. The figure shows that the collected mixed PPG signal includes substantial in-band noise, i.e., crossover and overlaps of signal and motion artifacts frequencies, because of which, class filtration does not perform well.

Motivated with this example, and since the ground truth for the source-separated signal (fetal PPG in this example) is not available in such health monitoring applications, we generate a synthesized dataset to evaluate the effectiveness of our signal separation method. Key features of the synthesized dataset, such as the pulsation and respiration rates are derived from available *in vivo* measurements. As another validation step using animal experimental data for which, the ground truth signal sources are unknown, we demonstrate improved performance of a downstream task, specifically fetal blood oxygen saturation estimation, when our proposed signal separation method is employed.

## 3 Related Work

Single-detector signal separation methods can be broadly classified into three categories: (1) Analytical component decomposition methods with or without consideration of the periodic behavior, (2) Deep learning methods that require ample datasets, and (3) Deep prior learning methods.

### Analytical methods:

**Empirical Mode Decomposition (EMD)** [9] and **Variational Mode Decomposition (VMD)** [2] (and their extension methods such as [1, 8]) decompose a time-domain signal into **Intrinsic Mode Functions (IMFs)**, representing different sources. EMD considers signal’s local extremas for extracting IMFs while VMD defines a variational problem assuming each source to have a compact frequency representation around a central frequency which tends to provide better frequency separation than EMD. **Non-negative Matrix Factorization (NMF)** [14] operates in both time and frequency domains. In time-frequency, it breaks down the 2-D matrix into two product matrices, which can be interpreted as distinct sources.

Presented for sound signals’ separation in time-frequency domain, **REpeating Pattern Extraction Technique (REPET)** [21] works under the assumption that a sound signal can be represented as sum of a repeating background with a varying foreground, where the proposed methodology identifies the repeating time–requency patterns over time and subtract them from the mixture to isolate the foreground. An extension of REPET, REPET-Extended, can adapt to changes in the repeating structure over time, providing better separation quality when the repeating parts of the signal are non-stationary.

### Data-intensive learning methods:

Leveraging the availability of extensive datasets in the audio domain, extensive research has been conducted on audio source separation using both supervised and unsupervised methods. Applied in the time-frequency domain, [10] employed deep clustering to learn embeddings for each spectrogram bin. These embeddings are then utilized to derive an optimal mask for separating sources in single-channel multi-speaker audio scenarios. Building on this, [15] enhanced music source separation by integrating the deep clustering method with traditional supervised learning. Their approach employs a dual-headed network that simultaneously performs clustering and ideal mask estimation. Following the groundbreaking success of U-Net neural architecture in biomedical image segmentation [23], the U-Net structure was adopted in supervised audio source separation by [11]. The U-Net’s multi-scale structure effectively captures both high-level features and fine details, facilitating the learning of ideal masks for singing voice separation in the time-frequency domain. Addressing the inherent limitations of time-frequency domain separation, such as phase reconstruction challenges, task-specific fine-tuning of STFT parameters, and latency issues with high-resolution STFT, [28] modifies the U-Net architecture for one-dimensional time-series (audio wave) input. Further innovating in this area, [16, 17] introduce a signal separation method in time domain with focus on real-time speech application. They introduce two network variations, one leveraging LSTM units and the other convolutional layers, which are adept at learning representations of separated audio signals from mixed speech inputs. The output representations are then converted back into waveform format using a linear decoder. Ref. [22] focus on enhancing high-frequency accuracy of separated signals by reconstructing high-frequency components by comparing input mixed spectrograms and separated signal spectrograms.

To address the limitations of supervised training methods that rely on synthesized data with unrealistic/limited mixing models, researchers have explored unsupervised methods to achieve higher generalizability without access to isolated sources. Ref. [18] proposes an unsupervised generative adversarial network method that models the distribution of individual sources within mixed signals. Ref. [33] introduces a novel unsupervised approach that separates signals with unknown number of sources. The training examples are sum of two real-world single-channel mixed data, and the model is trained to find underlying sources by comparing their selective sum with the original mixed data. Taking this further, [30] introduces an innovative unsupervised framework for audio-visual on-screen sound separation, capable of isolating on-screen sounds. This approach does not restrict the sound domain to specific classes, accommodating variable numbers of sources, and eliminating the need for labels or visual segmentation.

All these methods rely on availability of large datasets which is not applicable in many real-world applications where excessive data acquisition is costly and impractical.

### Deep Priors:

In their pioneering work, Ulyanov et al. demonstrated that the structure of a generative convolutional deep neural network, $f_{\theta }$, can be used as a prior for image denoising, in-painting and super-resolution tasks using only one sample in the dataset [31]. The network receives a random vector, z, as input, and is subsequently trained to generate an output that is *similar* to a *single*, given noisy, low-quality or masked image, $x_{0}$. Therefore, without requiring a large dataset to train the network on, the training method minimizes the distance between the neural network’s output and the input image. More formally, the training minimizes the cost function ${\text{min}}_{\theta }E\left(f_{\theta }(z);x_{0}\right)$, where E is a task specific term. The cost function of the in-painting task, which has also been used in this work, is presented in 1. The mask contains binary values, either 0 or 1, with a shape similar to the input image that conceals certain parts of the image from the cost function.


(1) $$E\left(x;x_{0}\right)=\|\left(x-x_{0}\right)⊙\text{mask}{\|}^{2}$$

Zhang et al. adopted this idea for audio processing by designing harmonic convolutions, and showed how time-frequency priors emerge from such neural networks [35]. They argue that the natural statistics of audio signals and their time-frequency spectrograms are different than images, and therefore, their prior design should differ. They use the patterns of harmonics in time-frequency plain to redefine neighborhood in frequency in their proposed deformable harmonic convolution. By their definition, the convolution output at each frequency is the weighted sum of input at integer multiples of that specific frequency. They further extend this definition to consider backward harmonic access by introduction of an anchor that multiplies integral multiples to ${\text{anchor}}^{-1}$.

Employing a deep convolutional prior technique, authors of [29] employ the auditory coherence and discontinuity feature of sound sources to separate the source signals using only a single-detector sound in time-frequency space. The technique utilizes two separate parallel networks per source that generate the signal and its silencer mask, mixing them together at their output to compare against the mixed input signal. However, this method is ineffective for separation of continuous quasi-periodic signals that inherently do not have silence gaps.

We have previously reported preliminary results that highlighted the promise of deep priors for quasi-periodic signal separation. This article substantially advances the preliminary work in [24, 25].

## 4 Proposed Methodology: Signal Separation via In-Painting of Resampled Masked Spectrograms

**Fig. 2.**
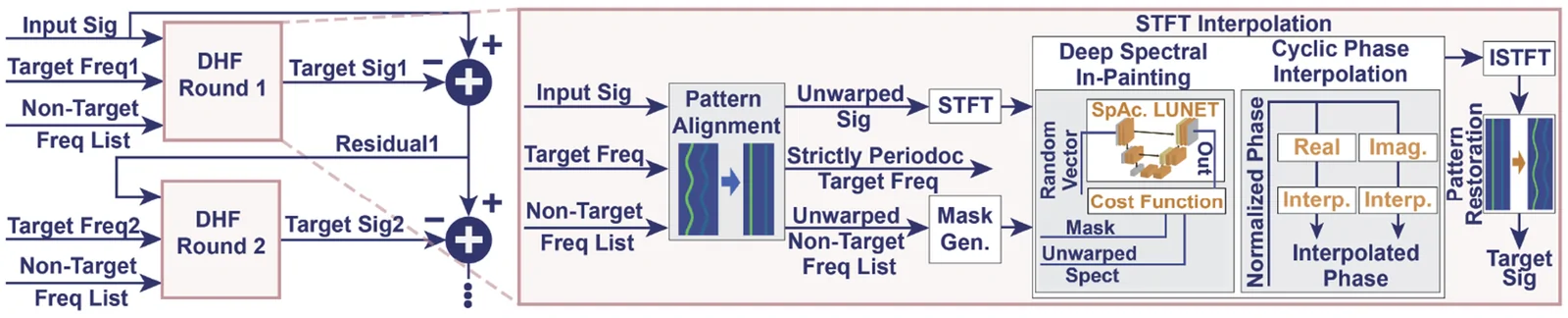
Overview of the proposed approach to signal separation. An application-specific neural network in-paints the masked spectrogram of the mixed signal, using a random input vector as input, thus, only one data trace is needed.

We propose an iterative method for signal separation in multi-source quasi-periodic signals using only a single mixed-signal in the dataset, named DHF. Each round begins by selecting the signal to be separated, followed by a proposed deep prior learning procedure for masking and in-painting the STFT. The mask conceals all significant spectral components of sources other than the selected one. In-painting interpolates the masked regions of the selected signal, targeting amplitude and phase separately, to recover values during spectral overlaps and crossover. The structural prior used for spectrogram in-painting consists of two complementary components: a signal pattern aligner and a specialized convolutional neural network that we call the Spectrally Accurate Light U-Net (SpAc LU-Net). The pattern aligner resamples and unwarps the mixed signal, transforming it into a space where the selected source is strictly periodic (holding a steady fundamental frequency of 1 Hz). Given the strict periodic pattern over time and the harmonic patterns in spectrum, SpAc LU-Net defines the neighborhood in time and frequency by dilation and accessing integral multiples of 1hz distance from target bin, respectively. Concurrently, phase interpolation is performed by separately interpolating the real and imaginary parts of each frequency bin’s phase over time, and then recalculating the interpolated phase. The spectrogram in-painting results, joined with the interpolated phase information, are then passed through the **inverse short time Fourier transform (ISTFT)** and transformed back to the original space (referred to as pattern restoration) to deduce the separated signal time-serie. After subtracting the separated source from the mixed signal, the residual is used for subsequent rounds of signal separation. Figure 2 sketches an overview of the proposed method, where each DHF block corresponds to one round of signal separation.

### 4.1 Target Pattern Alignment

**Fig. 3.**
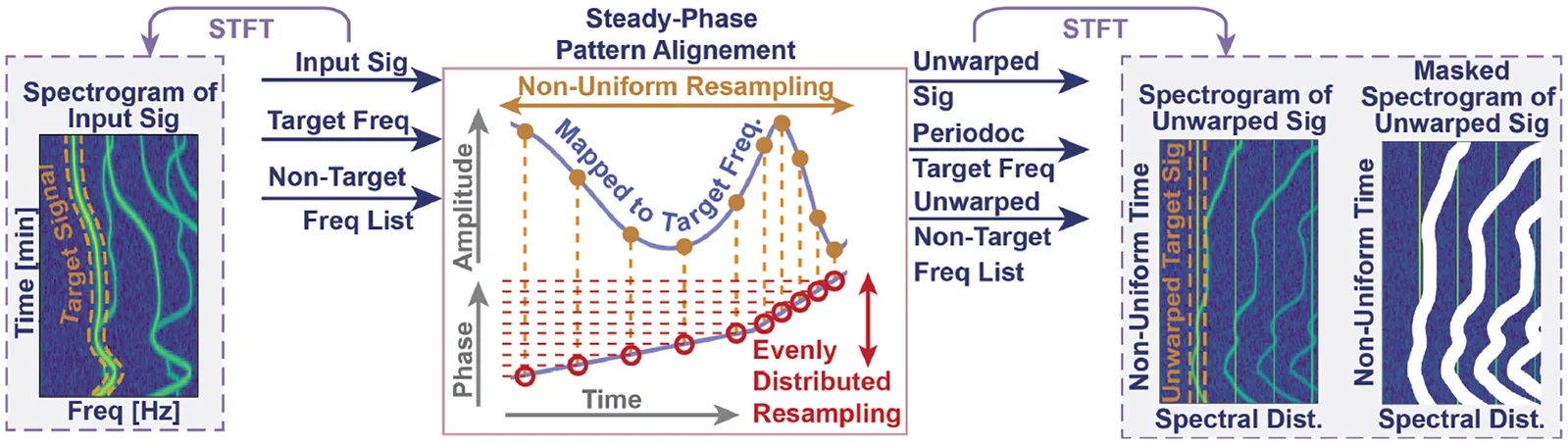
The pattern alignment procedure involves resampling of mixed signal from the given time-triggered sample stream to a phase-triggered sample stream, with respect to instantaneous frequency of a specific target signal. As a result of this invertible transformation, the target source will have a constant frequency in the transformed domain (unwarped signal).

In each round of signal separation, specific to a chosen target source, we resample the mixed signal, X, and unwarp it with respect to the fundamental frequency of the target source, $f_{ts}[n]$, locking a constant fundamental frequency of 1 Hz for that particular source in the unwarped signal, $X^{\prime }$. In other words, the adaptively resampled signal will pattern a strictly periodic target signal with constant frequency, and a varying amplitude over time. Therefore, the rate of samples in time is no longer uniform and varies based on original target frequency pattern. Instead, the samples will be evenly distributed in phase, for that reason, from this point forward we call the resampled space the steady-phase space, in contrast to the original steady-time space. Figure 3 depicts the resampling approach and the spectrogram view of an example signal before and after pattern alignment. The pattern alignment unit also provides the spectral pattern of non-target sources in the steady-phase space, which is used for masking those signals during spectrogram in-painting and phase interpolation.

The steady-time space (before resampling) is defined in Equation 2. With a full period of the quasi-periodic target source showing a phase change from 0 to 2π, Equation 3 computes the unrolled phase at each evenly spaced sampling time, t[n].


(2) $$(t[n],X[n]),\;\text{where}\;t[n]=n\Delta t=\frac{n}{F_{s}}$$

(3) $$\Phi [n]=2\pi \sum_{i=1}^{n}f_{ts}[i]\Delta t=\frac{2\pi }{F_{s0}}\sum_{i=1}^{n}f_{ts}[i]$$

Fluctuations in the fundamental frequency result in a variable phase interval between successive samples. To unwarp to a constant fundamental frequency of 1Hz and transforming the signal to the steady-phase space, the phase distance between every pair of consecutive samples of $X^{\prime }$ must remain constant, as indicated in Equation 4. The steady-phase signal $X^{\prime }$ is calculated by two sequential interpolations, detailed in Equations 5–6, first finding the timestamps of the evenly distributed phase intervals, $t^{\prime }[m]$, and then the mixed signal values at those timestamps, $X^{\prime }[m]$.


(4) $$\forall m,\Phi^{\prime }[m]-\Phi^{\prime }[m-1]=\frac{2\pi }{F_{s}^{\prime }}$$

(5) $$(\Phi [n],t[n])\to \left(\Phi^{\prime }[m],t^{\prime }[m]\right)$$

(6) $$(t[n],X[n])\to \left(t^{\prime }[m],X^{\prime }[m]\right)$$

To effectively filter out non-target signals within the steady-phase space, it’s also necessary to compute the transformed frequencies of any interfering sources. This involves resampling similar to Equation 6, while using the ratio of the non-target frequency to the target frequency time-series (for each interfering source) as the input signal.

The steady-phased signals can be transformed back to the steady-time space by interpolation from the resampled timestamps, $t^{\prime }[m]$, to the original timestamps as outlined in Equation 7.


(7) $$\left(t^{\prime }[m],X^{\prime }[m]\right)\to (t[n],X[n])$$

### 4.2 Application-Specific Neural Network Architecture

We leverage specific features of the application, or *priors*, in design of a deep neural network architecture. In particular, the steady-phase (resampled) signal spectrogram encapsulates critical features, which are considered in deep neural network architecture. These features outline the spatial neighborhood in both the time and spectral dimensions. Locking a constant fundamental frequency for the target source suggests that, to access preceding and subsequent amplitudes of that source over time, the convolutional kernel can exclusively examine the same frequency bin via dilation in time, $D_{t}$. Neighbors in frequency are then defined as integral multiples of 1Hz distance from the current frequency, $D_{f}$. The convolution operation between the pattern-aligned (strictly periodic target signal) time-frequency spectrogram, $S[\overset{ˆ}{\omega },\overset{ˆ}{t}]$, and the kernel K with H total harmonics at any specific frequency location, $\overset{ˆ}{\omega }$, and time, $\overset{ˆ}{t}$, is formulated in Equation 8.


(8) $$\left(S*K)[\overset{ˆ}{\omega },\overset{ˆ}{t}]=\sum_{k=1}^{H}\sum_{t=-T}^{T}S\left[\overset{ˆ}{\omega }+kD_{f},\overset{ˆ}{t}-D_{t}t\right]K[k,t\right]$$

**Fig. 4.**
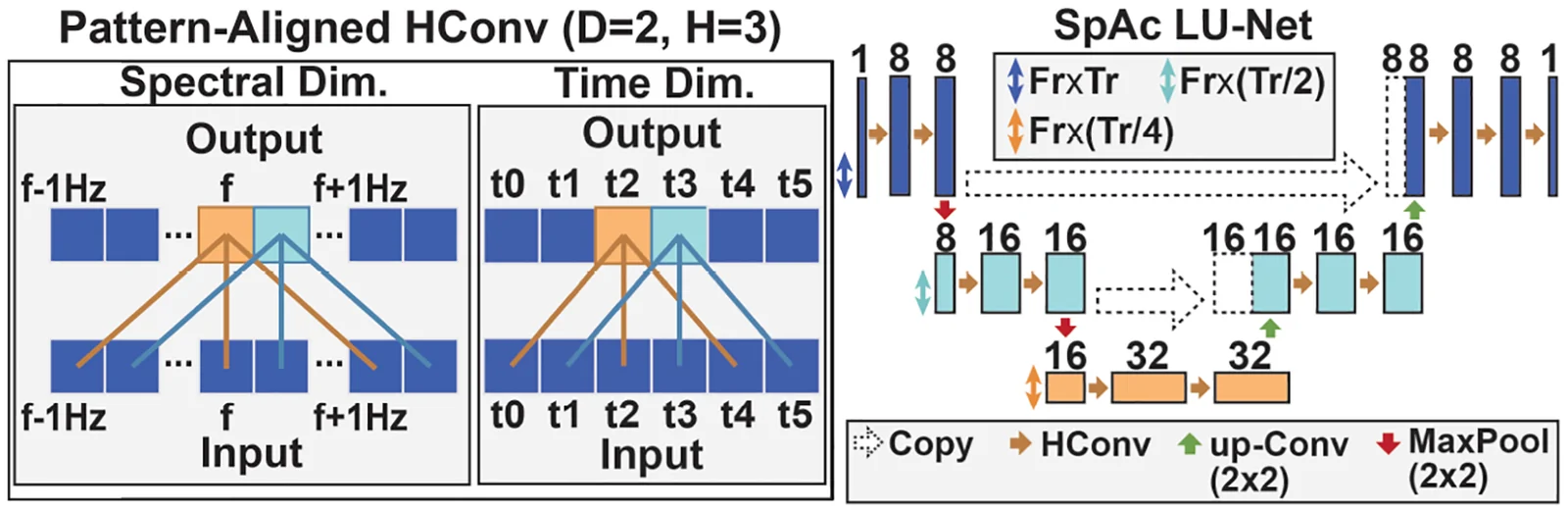
Dilated harmonic convolution and the structure of Spectrally-Accurate (SpAc) LU-Net.

We have adapted the U-Net architecture [23] as the foundation of our neural network structure, while substituting standard convolution kernels with dilated harmonic convolutions. Moreover, two design principles are maintained for harmonic integrity. Firstly, pooling in the frequency dimension is prohibited, ensuring the size of this dimension remains unchanged throughout the network. Secondly, the dilation in frequency is set to 1Hz stride (only towards higher frequencies) to promote the strict distance of the consecutive target’s harmonics after pattern alignment, while suppressing noise and interference. Figure 4 depicts the proposed Spectrally-Accurate (SpAc) LU-NET structure and the function of the proposed dilated harmonic convolution in time and frequency dimensions.

Given the application-specific setting of the spectrogram’s resolution in both time and frequency, the parameters $D_{t}$ and $D_{f}$ must be adjusted accordingly. The parameter $D_{t}$ determines the temporal reach, which should be calibrated based on the prior knowledge on the dynamics of the target signal. A broader temporal reach is indicative of a more relaxed prior setup, enhancing smoothness, whereas a narrower reach is advantageous for capturing signals with rapid amplitude changes. In the context of the SpAc LU-Net architecture, the inclusion of each max-pooling layer doubles the temporal reach. Consequently, the adjustment of $D_{t}$ must take into consideration the effect of two max-pooling layers within the proposed SpAc LU-Net framework.

### 4.3 Signal Separation via Spectrogram In-Painting

We use masking and in-painting to separate sources in quasi-periodic mixed signals. At each round, we use a binary mask to conceal all significant harmonics of non-targeting sources from the cost function. The in-painting cost function is similar to the original proposition by [31] for image in-painting applications. The selective visibility of the mask allows the optimizer to focus solely on the target source, in-painting the missing cross-overs and overlaps to retrieve the desired source.

Equation 9 presents the in-painting cost function, wherein $S_{\text{mixed}}$ and $S_{\text{out}}$ represent the mixed spectrogram and the neural network’s output spectrogram, respectively.


(9) $$E\left(S_{\text{out}};S_{\text{mixed}}\right)=\|\text{mask}*\left(S_{\text{out}}-S_{\text{mixed}}\right){\|}^{2}$$

**Fig. 5.**
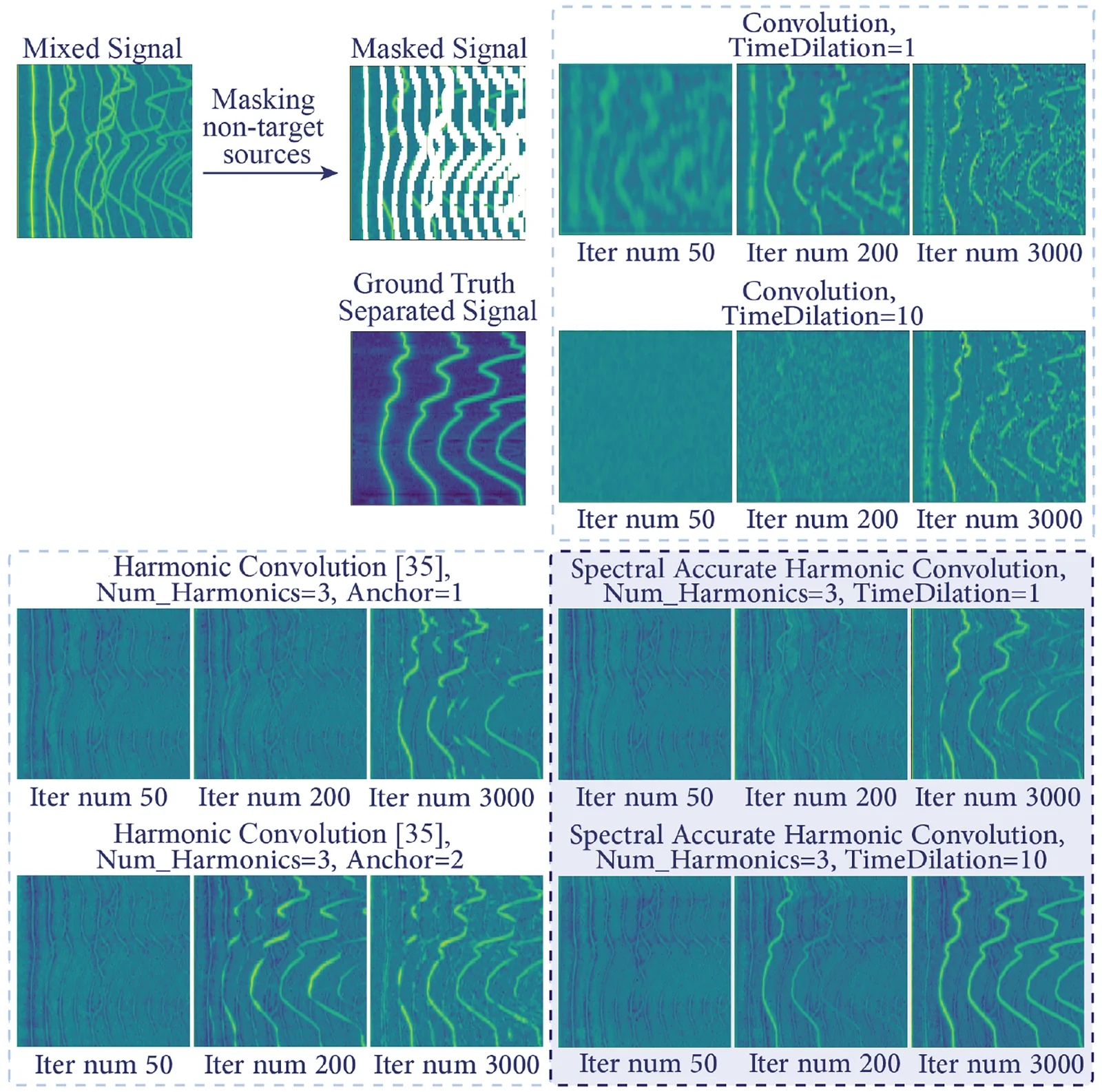
Example performance comparison of different convolutional kernels with the same network structure.

Figure 5 illustrates the effectiveness of the proposed neural network structure, SpAc LU-NET with harmonic convolutions, in its role as implicit priors for learning quasi-periodic time-frequency patterns. The figure provides a comparative analysis of learning outcomes from different convolution kernels and network configurations receiving the same masked image. A comparison between conventional convolution kernels and harmonic convolution layers reveals the repetitive vertical frequency patterns, which are noticeable even at the initial stages of learning. The proposed method extends and improves upon the baseline harmonic convolution design introduced in [35] in two major ways. First, the proposed approach further constrains the harmonic convolution function to enforce physically accurate consistency and enhance noise-reduction capability. This is achieved by allowing only forward harmonic access, eliminating inaccurate backward harmonic associations, and removing max-pooling operations in the frequency domain. Second, and more importantly, the introduction of the steady-phase transformation results in a deterministic structural alignment in frequency space by enforcing a fixed-distance placement of the target harmonics. This enables the use of a more restrictive prior with fixed spectral dilation in the harmonic convolution function. As a result, the proposed adaptive harmonic prior surpasses traditional methods by minimizing interference from non-target frequencies while facilitating access to consecutive harmonics. Since consecutive harmonics typically contain more energy than isolated frequency bins at integer multiples, this design offers substantial benefits for in-painting higher-frequency components. Consequently, the proposed spectrally accurate design demonstrates significant noise reduction compared to the state of the art. This improvement is particularly pronounced when larger time-domain dilations are employed, which further encourage the persistence of vertical target patterns over time.

#### Stable Training under Interference:

Multiple parameters can affect the performance of DHF signal separation. Two key factors are target’s masked duration relative to the rate of amplitude change over time, and the target’s energy ratio relative to noise or interfering signals. Varying the overlap duration alters the minimum required time dilation for accessing clean target signal that is visible during learning and can guide the optimization to convergence. More specifically, when convolution kernel is applied to the center of the longest overlap, time dilation can be adjusted to access clean data before and after an overlap. However, since the network structure acts as an implicit prior, time dilation should be limited by a maximum threshold set based on the rate of signal variation over time. In presence of a long overlap duration, where the minimum required dilation to access unmasked data exceeds the maximum threshold, a lower dilation setup can impair optimization stability and convergence. Another factor affecting DHF performance is the target’s energy ratio to noise or interference, which can also undermine stability and convergence if this ratio is small. In such cases, incorporating a secondary term in the cost function to enforce an explicit prior can aid convergence.

To further enhance optimization convergence, expanding the cost function to include some measure of the variation of energy per target’s harmonic over time is beneficial. Owing to the strict periodic harmonic pattern of the target after pattern alignment, penalizing the cost function with squared energy variation at select frequency bins, representing specific signal harmonics, is advantageous. This term helps enforce smooth energy variation in masked areas with long duration. Furthermore, it suppresses sudden energy jumps in the unmasked spectrogram regions due to noise and interference. Energy variation of the kth harmonic at time stamp $\overset{ˆ}{t}$ and time increment Δt, assuming $2w_{h}+1$ bins carry the overall energy of the select harmonic at the specified time stamp, is expressed in Equation 10. Equation 11 presents the proposed squared variation term, where $T_{S}$ and $n_{h}$ represent the spectrogram time duration and number of considered harmonics.


(10) $$V(\overset{ˆ}{t},k)=\Delta_{\overset{ˆ}{t}}\left[\sum_{w=-w_{h}}^{w_{h}}S_{\text{out}}\left(kD_{f}+w,\overset{ˆ}{t}\right)\right]$$

(11) $$SqV=\sum_{\overset{ˆ}{t}=1}^{T_{S}}\sum_{k=1}^{n_{h}}[V(\overset{ˆ}{t},k){]}^{2}$$

However, introducing squared variation term to the cost function too early can misdirect learning and trap optimization in a local minimum, where a relatively low energy, close to background levels, is assigned to target signal’s frequency bins. To mitigate this issue, we apply a dynamic weight to the squared variation term that increases over optimization iterations. Equation 12 explains the dynamic weight formulation, where $a_{tv}$ and $b_{tv}$ control the slope and position of the weight increase in relation to the optimization iteration number, $n_{i}$.


(12) $$L_{tv}\left(n_{i}\right)=\frac{w_{max}}{1+e^{-a_{tv}\left(n_{i}-b_{tv}\right)}}$$

By this definition, the proposed cost function is presented in Equation 13.


(13) $$\text{Cost}=w_{0}E\left(S_{\text{out}};S_{\text{mixed}}\right)+L_{tv}\left(n_{i}\right)SqV\left(S_{\text{out}}\right)$$

### 4.4 Cyclic Phase Interpolation

**Fig. 6.**
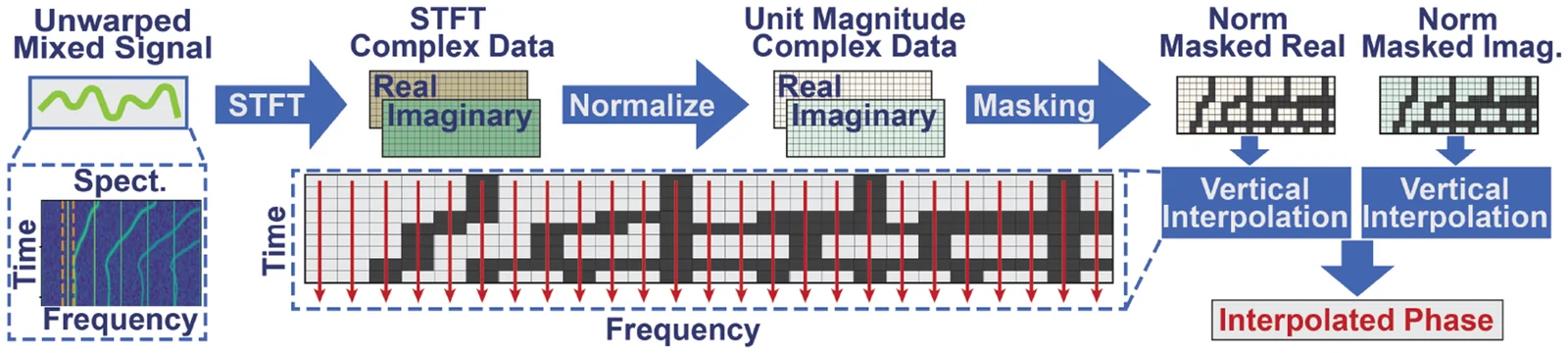
Cyclic phase interpolation applied on the pattern-aligned (unwarped) time-frequency spectrograms.

Phase interpolation of the STFT data is conducted independently from the spectrogram in-painting. Figure 6 illustrates an overview of the proposed phase interpolation procedure. The phase interpolation input signal is the pattern-aligned mixed signal, unwarped around the fundamental frequency of a specific target, and the generated mask that marks interpolation areas due to interference. Applying STFT transform, the initial complex data contains both amplitude and phase information. To omit the amplitude information, we apply amplitude normalization on the STFT data, setting an amplitude of one for every complex data point. Utilizing the previously generated mask, we then estimate the phase during overlaps and crossovers through interpolation. Owing to the strictly periodic patterns in pattern-aligned time-frequency space, we interpolate each frequency bin over time separately, and concurrently with other frequency bins. For each bin, we interpolate both the real and imaginary components of the normalized masked data. Since a complex phase $e^{j\phi }=\text{cos}\left(\phi \right)+j\;\text{sin}(\phi )$ evolves continuously in sin(ϕ) and cos(ϕ), interpolating the real and imaginary components avoids the artificial discontinuities introduced when the phase angle ϕ is wrapped modulo 2π. Subsequently we calculate the interpolated phase by integrating these results.

### 4.5 DHF Algorithm Implementation

To enhance the reproducibility and clarity of the repetitive signal separation in DHF, the implementation process is further detailed in Algorithm 1. In this algorithm, the sections where output representation is in time or spectral domain are marked with blue and red markers respectively.

## 5 Performance Evaluation

We evaluate the performance of the proposed method, and compare it with a number of competing methods, using both synthesized and *in vivo* TFO datasets. Initially, we delve into the specifics of the synthesized dataset we created and present our empirical findings on the synthesized data. Following this, we discuss experiments with *in vivo* data acquired through experiments involving pregnant ewe with hypoxic in-utero fetal lambs.

### 5.1 Synthesized Data

We have created a tool for generating and characterizing task-specific synthesized quasi-periodic time series data, g[n], with a sampling frequency of $F_{s}$ and a total of np complete periods. Each period is characterized by the desired input function per period, I(t), time duration per period list, D, and amplitude per period list, A, as formalized in Equations 14 to 16. The signal’s shape and intensity for these settings can be specified either in CSV format or using an input image. W(t) in Equation 16 represents a windowing function, which enforces the signal h to have equal values at the boundaries, and thus, the periodic waveform to be smooth.


(14) $$D=\left[d_{1},d_{2},d_{3},\ldots ,d_{np}\right]$$

(15) $$A=\left[a_{1},a_{2},a_{3},\ldots ,a_{np}\right]$$

(16) h(t)=I(t)W(t),t∈[0,2π)

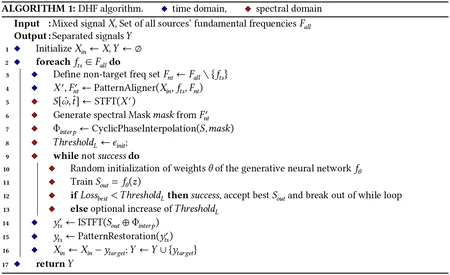


Employing the phase, ϕ[n], defined earlier in Equation 5, the phase at each sampling time is computed by feeding the $f_{ts}$ values derived from the inputted time duration list and sampling frequency (Equation 17). Next, g[n] is calculated using the windowed function and the phase information, and finally, a Gaussian noise term is added to the synthesized waveforms.


(17) $$f_{ts}=\text{repeat}\left(\frac{1}{D},D⊙F_{s}\right)$$

(18) $$a_{ts}=\text{repeat}\left(A,D⊙F_{s}\right)$$

(19) $$g[n]=a_{ts}[n]h\left(\left(\phi [n]+\phi_{0}\right)\%2\pi \right)$$

We have generated 5 distinct synthesized mixed quasi-periodic signals with sampling frequency of 100Hz for TFO application. Each mixed signal has 2 or 3 sources for respiration, maternal pulsation, and fetal pulsation. The respiration PPG shape is extracted from sheep experimental data (discussed in 5.3) after filtering out other dynamics [4, 32]. The pulsation PPG shape for each case is randomly extracted from MIMIC-IV dataset [7, 12].

**Fig. 7.**
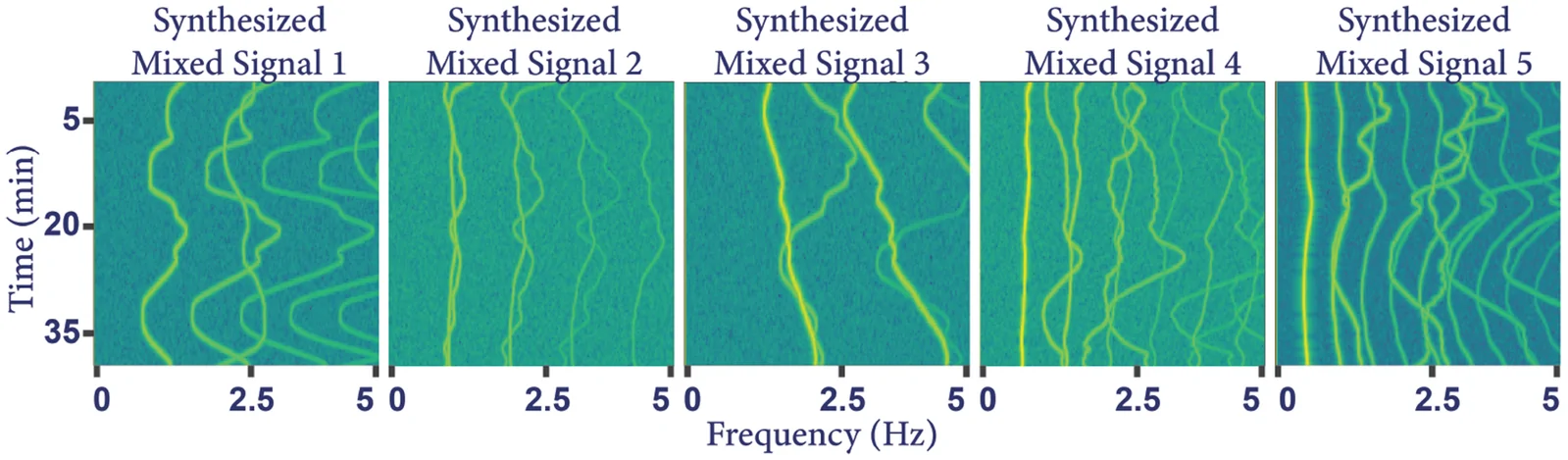
Spectrogram of the synthesized mixed signal traces generated for signal separation.

The spectrogram of the generated mixed signals is visualized in Figure 7. Key characteristics of the synthesized signals are further summarized in Table 1. Mixed Signals 1 to 3 each have two sources (maternal pulsation and fetal pulsation). The sources in Mixed Signal 1 show interference in the second harmonic of the target source, while Mixed Signal 2 has interference on the first harmonic. In Mixed Signal 3 the second source has less than 10% of the dominant source’s amplitude. Mixed Signals 4 and 5 each contain three source signals (respiration, maternal pulsation, and fetal pulsation), with differences in overlap duration and complexity. Similarly, the amplitude of their third source is not more than 10% of the first source.

### 5.2 Experimental Results on Synthesized Data

**Fig. 8.**
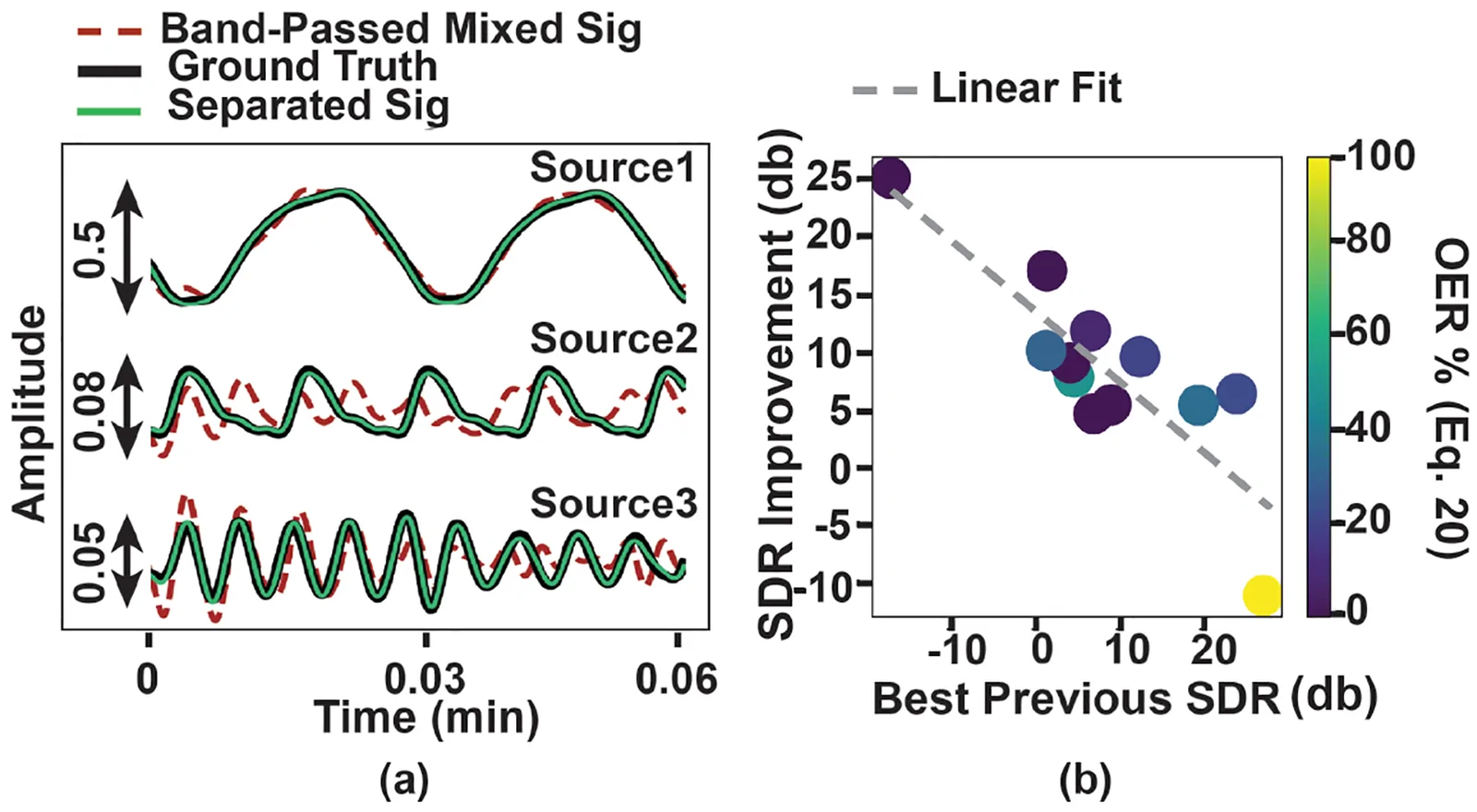
(a) Example signal separation performance on a short snippet of synthesized data. (b) SDR improvement (in dB) versus Spectral Masking (the best performing previous method) showing higher improvement in cases that are more difficult for existing techniques, highlighting the gap addressed by the proposed method.

Figure 8(a) visualizes an example signal separation using both DHF and a simple band-pass filter on a snippet of MSig5. The results reflect a very close decomposition of all three sources by DHF. We further explore the performance of DHF in comparison to existing signal separation methods when applied on the generated mixed signals.

#### Metrics:

To quantify the performance of signal separation methods, we utilize **Signal to Distortion Ratio (SDR)** and **Mean Squared Error (MSE)**. SDR is analogous to SNR, with the difference that the “noise” is made up of the difference between the estimated and the reference value of a source, thus, the higher the SDR, the better. MSE captures the average energy of the difference between the estimated and the reference source, and thus, the lower the MSE, the better. For averaging MSE values across testbenches, we use geometric averaging, whereas for SDR values, which are reported in logarithmic dB scale, we use arithmetic averaging in their original linear scale, before converting back to logarithmic dB scale.

To further analyze the efficacy of DHF in various scenarios, we introduce three additional metrics: the Total Energy ratio, TER, the Masked Energy Ratio, OER, and the Target Mask Duration Ratio, TMDR, as detailed in Equations 21 and 22.

TER presents the ratio of target signal energy to mixed-signal energy, while OER captures the ratio of the masked target source energy to total overlapping mixed-signal energy in the regions that the mask overlaps with the target source. In other words, OER is a ratio between zero and one, which captures the percentage of masked mixed signal energy in the overlap regions that is due to the target source. That is, lower OER values indicate that the source is fairly weak in comparison to the overlapping signals in the masked regions. Finally, TMDR, defined as the ratio of target mask duration to overall signal duration, aims to provide a measure of target signal’s temporal visibility (remaining unmasked regions) for in-painting. Higher TMDR values correspond to more difficult cases in which, the target source is mostly masked throughout the trace.


(20) $$\text{TER}=\frac{\text{Total Target Energy}}{\text{Total Mixed Energy}}$$

(21) $$\text{OER}=\frac{\text{Masked Target Energy}}{\text{Masked Overlap Mixed Energy}}$$

(22) $$\text{TMDR}=\frac{\text{Target Signal}\prime \text{s Masked Duration}}{\text{DataTrace Duration}}$$

#### Experiment Setup:

Mixed signals (MSig) are low-pass filtered with a cutoff frequency of 12 Hz, and their spectrograms are generated with window length, and stride of 60s and 15s, respectively. A dilation parameter of either 13 or 15 is used for the specific data at hand. Let the longest masked duration of the target component in the unwarped, non-uniform time-frequency space be denoted by $M_{\text{max}}$. Empirical evaluation indicates that improved noise-reduction performance is achieved when the time dilation $D_{t}$ satisfies $D_{t}\geq D_{t,\text{min}}=\left\lfloor \frac{M_{\text{max}}}{2}\right\rfloor +1$. This lower bound ensures access to sufficient unmasked context around the maximum interference duration in the target signal. At the same time, $D_{t}$ must remain small enough to preserve sensitivity to amplitude variations of the target signal. Balancing these considerations, we select $D_{t}$ based on the average value of $D_{t,\text{min}}$ observed across the dataset, resulting in $D_{t}=13$ or 15 for the application considered in this work.

Some signal separation algorithms, such as **Independent Component Analysis (ICA)** and **Principal Component Analysis (PCA)**, don’t accommodate single-detector signal separation.

As a result, we excluded them from our study. We compare against six previous signal separation methods, EMD [9], VMD [2], NMF [14], REPET and REPET-Extended [21], and spectral masking [6]. The comparison results are presented in Table 2.

#### Discussion:

The results in Table 2 highlight that the DHF method achieves an approximate 106% (3.15db) improvement in SDR, and 85% reduction in MSE on average, compared to the best performing competing signal separation method, i.e., Spectral Masking. Particularly for sources whose amplitude is smaller than 10% of the dominant signal amplitude (such as mixed signal 3 source 2, mixed signal 4 source 3, and mixed signal 5 source 3), DHF demonstrates remarkable effectiveness. For these three low signal strength scenarios, DHF shows 5.76db and 93% average SDR and MSE improvement over the best prior method.

In Figure 8(b), we visualize a granular analysis of the SDR improvements achieved with DHF compared to Spectral Masking, the best performing competing method. The results indicate that DHF fills a critical gap, as it particularly excels in scenarios where existing methods fail. The figure employs a heatmap to illustrate the relationship between OER, best performance by a competing method, and performance improvement due to the DHF. Previous methods tend to struggle in low OER cases (darker dots), attempting to isolate low-energy signals from strong overlapping interference. DHF notably outperforms its predecessors in such cases.

Assuming knowledge of the fundamental frequency of each source gives our proposed technique an edge over previous approaches that do not incorporate this assumption. To evaluate the effectiveness of various signal separation methods, including this critical piece of information, we first apply a mask to retain only the target frequency signal, albeit with remaining overlaps and crossovers. Subsequently, we apply each signal separation method to this pre-processed data. The results, as shown in Table 3, indicate that while some prior methods show some improvements after preprocessing, DHF consistently outperforms the competition, keeping the aforementioned margin with respect to the best existing method.

#### Low Latency Separation via Short Time Frames:

The baseline version of DHF, as presented, works on spectrogram of a given data trace, regardless of its duration. Real-world health monitoring applications in which, response latency is important, demand signal separation using a constrained time frame. To address this topic, we evaluate the performance of DHF when applied to shorter segments of data, specifically within the 2 to 5-minute frames. Such an investigation not only mirrors the practical need for applications that demand low latency response, but also highlights the robustness and adaptability of our methodology to operate under a more stringent constraint on data availability.

We employ a sliding window technique on each synthesized mixed dataset to evaluate the performance of DHF under data traces of constrained duration. We experimented with two variations of the sliding window setup: firstly, a 5-minute window duration with 2.5-minute sliding steps, generating spectrograms with a 30-second window size and a 5-second stride; and secondly, a 2.5-minute frame duration and 2.5-minute sliding steps, where spectrograms were generated using a 20-second window size and a 3-second stride.

**Fig. 9.**
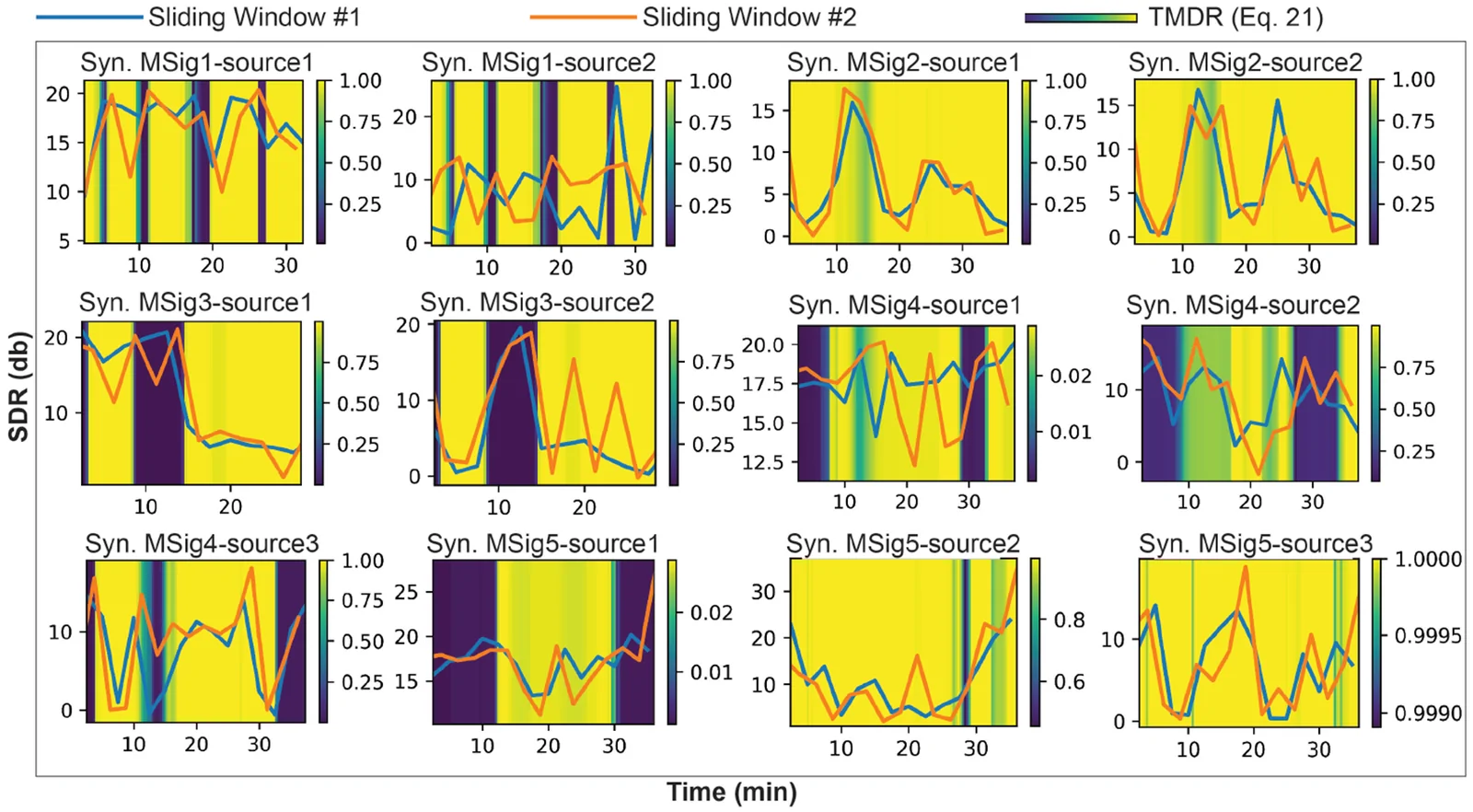
DHF performance study (SDR on y axis) when applied on five synthesized mixed signals with a constrained data frame of 5-minutes (sliding window #1) and 2.5-minutes (sliding window #2). The background heatmap highlights TMDR parameter, which captures target visibility in 1-minute sliding windows, where 0 and 1 refer to fully-visible and fully-masked respectively.

Figure 9 illustrates the performance of DHF in separation of each source, employing both sliding window configurations. The X and Y axis of the plots indicate time and SDR, respectively. The figures also include a color coded heatmap indicating the TMDR parameter, computed for each 1-minute frame duration. TMDR effectively captures the extent to which the target source is obscured by other interfering signals, ranging from 0 (fully visible) to 1 (completely masked).

The results underscore that SDR improves as the visibility of the target energy increases. Conversely, when the target becomes less visible, the behavior of SDR is commonly reduced with higher variation over time. A comparison between the two sliding window setups indicates that a smaller frame size leads to a more pronounced increase in SDR when the target energy is visible in the spectrogram, yet it also results in a faster decline in SDR when visibility diminishes. This nuanced observation underscores the delicate balance between frame size and the ability to maintain high separation performance in conditions where target visibility is challenged. In such scenarios, a dynamic frame duration that can extend the use of previous data can help with SDR improvement.

#### Impact of Interference Energy and Duration on DHF Performance:

**Fig. 10.**
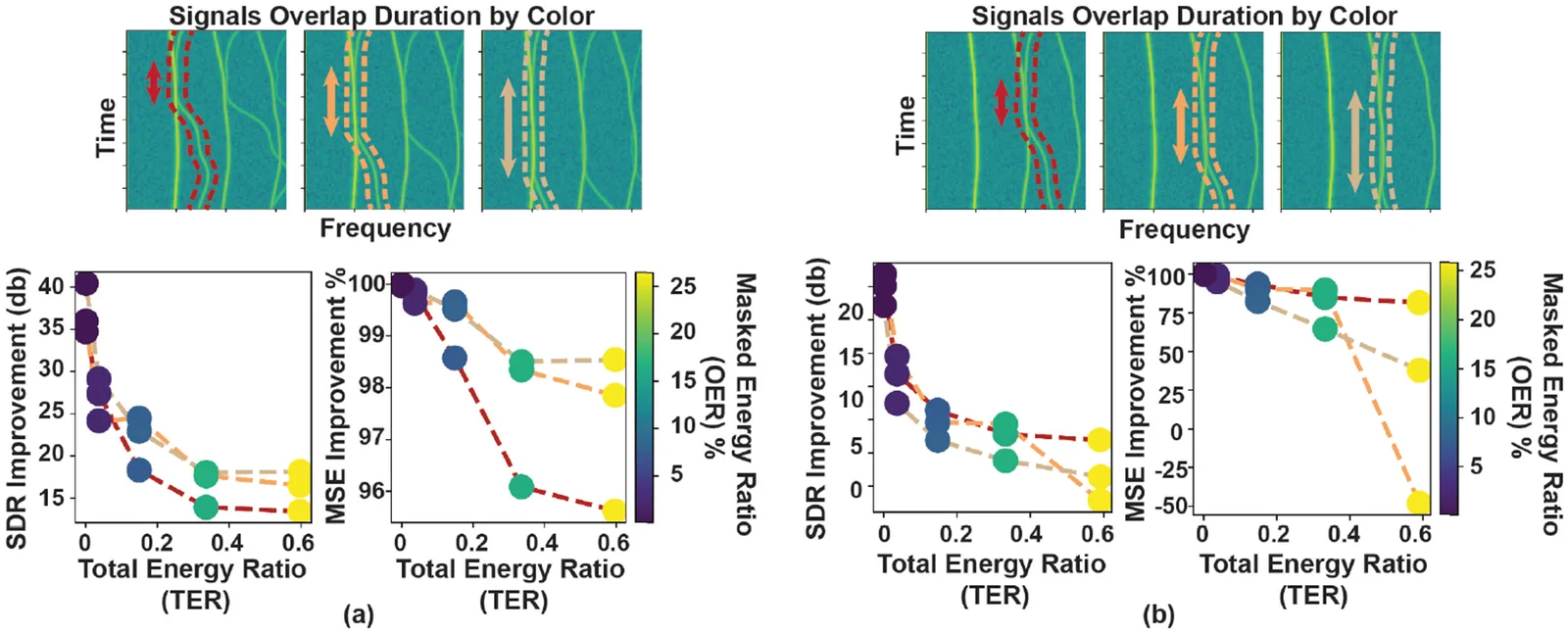
DHF performance improvement, in db, relative to the best previous method while varying overlap duration (X axis), and the energy ratio of the target signal in the masked regions (color coded dots), when (a) overlap is on the first harmonic. (b) overlap starts from the second harmonic of the target signal. The fundamental frequency of target signal is highlighted with dash lines in the top spectrogram image.

We examine the sensitivity of DHF’s SDR and MSE improvement (compared against best previous work) relative to two key variables, namely the overlap duration and Masked Energy Ratio, OER (Equation 21). Intuitively, we keep the target signal the same, but change the interference duration and energy levels. More specifically, we adjust the parameters with three distinct settings for overlap duration and five for TER. The top illustrations in Figure 10 outline our experimental setup and three dashline color codes for different overlap durations. Considering two extra setup in part a and b for overlap placement on first or second target harmonic, we introduce 30 new data points in this study. These data points are studied to model different application scenarios, such as non-invasive fetal monitoring in which, the fetal and maternal heart rate have different rates. The bottom plots in the figure show the SDR and MSE improvement for each data point relative to the best existing method.

We observe that when overlaps do not start from the fundamental frequency (part b), the performance of existing methods improve and, thus, we see relatively less improvement in SDR compared to the more difficult case, where fundamental frequencies overlap (part a). Focusing on the more challenging setup of part a, DHF shows a notable improvement in performance as the overlap duration increases, outperforming previous techniques that struggle to separate such sources accurately.

Focusing on OER variation, DHF maintains a steady performance gain at lower overlap energy ratios where interference effect is more pronounced and previous methods fail. As OER increases, signal separation becomes less challenging for existing methods, and thus, the performance gains observed with DHF become generally smaller, and sometimes more variable.

#### Computational Analysis:

Table 4 presents experimental results on the computational cost of the DHF algorithm. Ten repeated runs were performed on a single RTX A5000 GPU using the synthesized dataset described in Section 5.1. Although all signals span 45 minutes in the time domain, their representations in the transformed unwarped space vary in duration across datasets. This variation affects the network structure and, consequently, the in-painting execution time and memory usage. Analysis of individual in-painting steps shows execution times ranging from 17 to 47 seconds, with memory consumption well within the capacity of standard GPUs. Computational costs increase in later separation rounds, as the pattern aligner produces larger spectrograms when the target signal’s fundamental frequency increases. To optimize MSE for each signal, an application-specific threshold of ${\text{Threshold}}_{L}=0.001$ was applied at each in-painting step. In difficult cases involving high-energy interfering sources with lengthy overlap durations, this constraint may not be met where we iteratively relax the threshold by a 20% increase for the repeated fitting step (see Algorithm 1). For this dataset, the maximum runtime per source separation is 46.29 s, while the overall runtime for full source separation ranges between 80 and 140 seconds.

### 5.3 Results on *in vivo* Data from Experiments with Pregnant Sheep

**Fig. 11.**
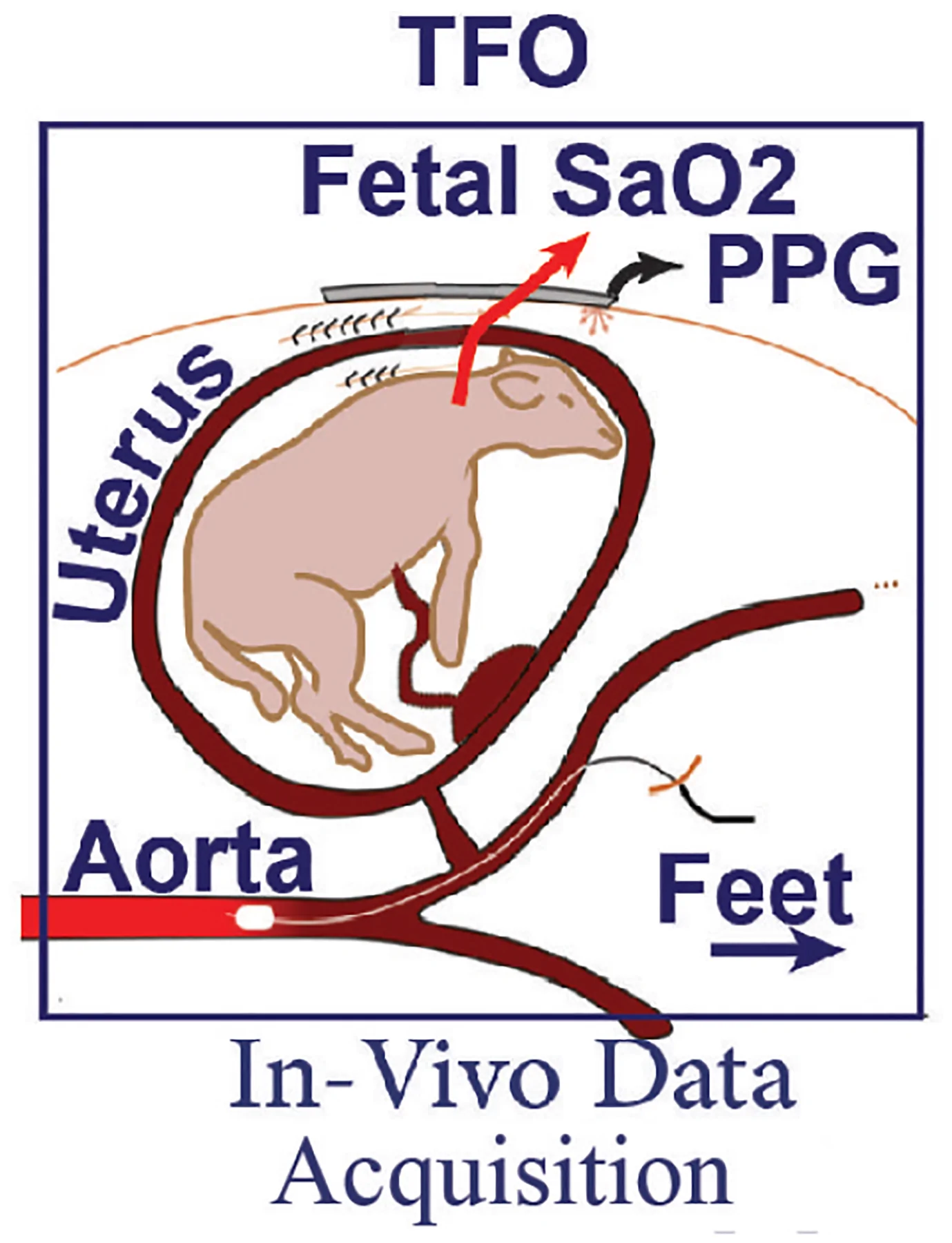
Sketch of the controlled in-utero fetal desaturation experiment used for data collection [4].

We study the impact of our approach on the accuracy of fetal blood oxygen saturation (fSpO_2_) estimation using a transabdominal photoplysmograph (PPG) dataset acquired from two term-pregnant ewes [4]. Figure 11 sketches the experimental protocol used in acquiring the data. The experiment involves instrumentation of an in-utero fetus to support blood draws through her carotid artery. An inflatable balloon catheter is placed in ewe’s aorta, using which the blood pressure to the uterus can be regulated, and controlled fetal hypoxia can be induced. The non-invasive TFO device placed on the ewe’s abdomen and collects data during the experiment.

**Fig. 12.**
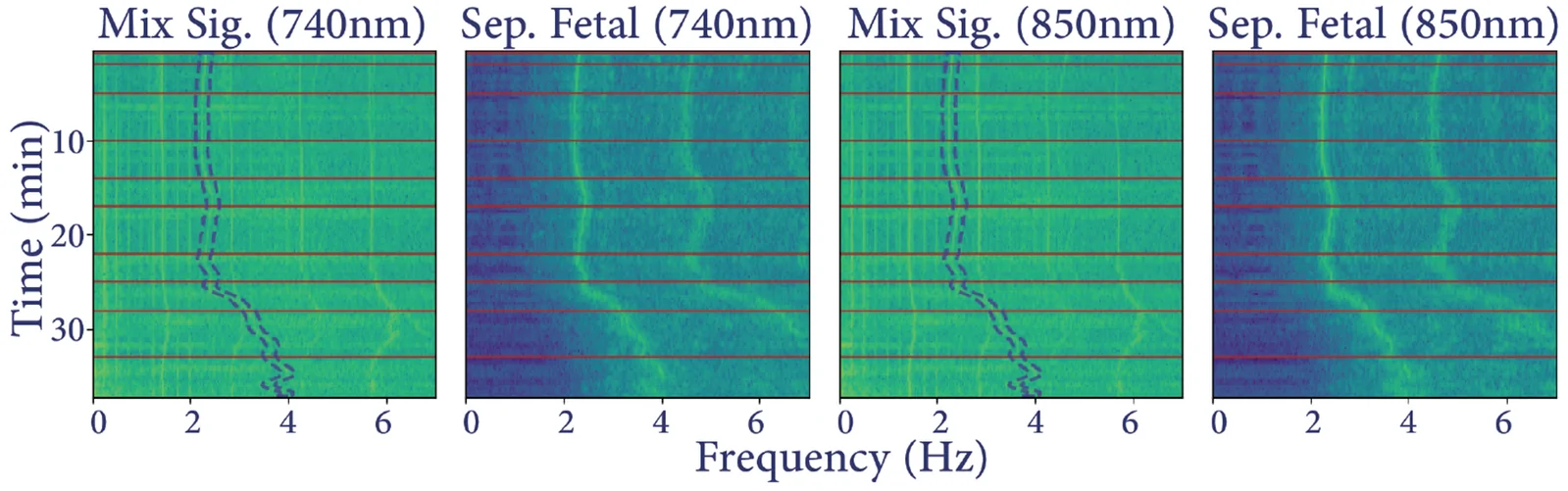
Performance of fetal signal separation in sheep data. Red horizontal lines mark the timestamps of fetal blood draws, and dashed vertical curves highlight the frequency of fetal lamb’s heart rate. As reference value of signal sources are unknown, performance of signal separation is quantified using a down stream task, namely, accuracy of estimating fetal blood oxygen saturation using the separated fetal signal.

The dataset consists of 40 minutes of continuous mixed PPG signals, at two 740nm and 850nm wavelengths, recorded from outside of the ewe’s abdomen. In addition, the dataset includes ground-truth fetal blood oxygen saturation readings, i.e. fSaO_2_, measured from arterial blood gas (ABG) analysis of fetal blood samples drawn at specific time points during the experiment. Figure 12 presents the measured mixed signal spectrograms for sheep 2 at the two 740nm and 850nm wavelengths, and the separated fetal signal at each wavelength using DHF method.

As the ground-truth fetal PPG signal, as would be observed from outside of the maternal abdomen, is not known, we use the correlation between estimated fSpO_2_ vs. measured fSaO_2_ readings (through ABG) as the evaluation criterion, for comparison the performance of the proposed signal separation algorithm vs. spectral masking method. We have compared against spectral masking since it showed the best performance among previous works using synthesized data, and is used in a state of the art method for fSpO_2_ estimation [32].

#### Fetal Arterial Blood Oxygen Saturation (fSpO_2_) Estimation:

After separating the fetal signal from mixed PPG signal collected in the aforementioned animal study using DHF, the method reported in [32] is used for estimation of fetal blood oxygen saturation through pulse oximetery (fSpO_2_). Specifically, the separated fetal PPG at a high level is used to calculate the fetal modulation ratio, R, given in Equation 23. Fetal modulation ratio is a measure of relative light absorption changes at two measurement wavelengths ($\lambda_{1}$ and $\lambda_{2}$), 0due to fetal arterial (pulsatile) blood flow. AC and DC parameters in the equation represent the dynamic and static parts of separated fetal PPG signal. Equation 24 shows the relationship between modulation ratio and the inferred fSpO_2_, where K is a constant and $v_{0}$ and $v_{1}$ are calibration parameters, which are adjusted to find the best fit for the given measurement setup.


(23) $$R=\frac{(AC/DC{)}^{\lambda_{1}}}{(AC/DC{)}^{\lambda_{2}}}$$

(24) $$fSpO_{2}=-k+\frac{1}{v_{0}+v_{1}R}$$

Equation 25 presents the employed linear regression method to obtain the values for parameters $v_{0}$ and $v_{1}$ of Equation 24 in which, fSaO_2_ values obtained from fetal blood samples are treated as reference fetal arterial oxygen saturation.


(25) $$Y^{\prime }=\frac{1}{SaO2+k}=v_{0}+v_{1}R,\;k=1.885$$

##### Discussion:

**Fig. 13.**
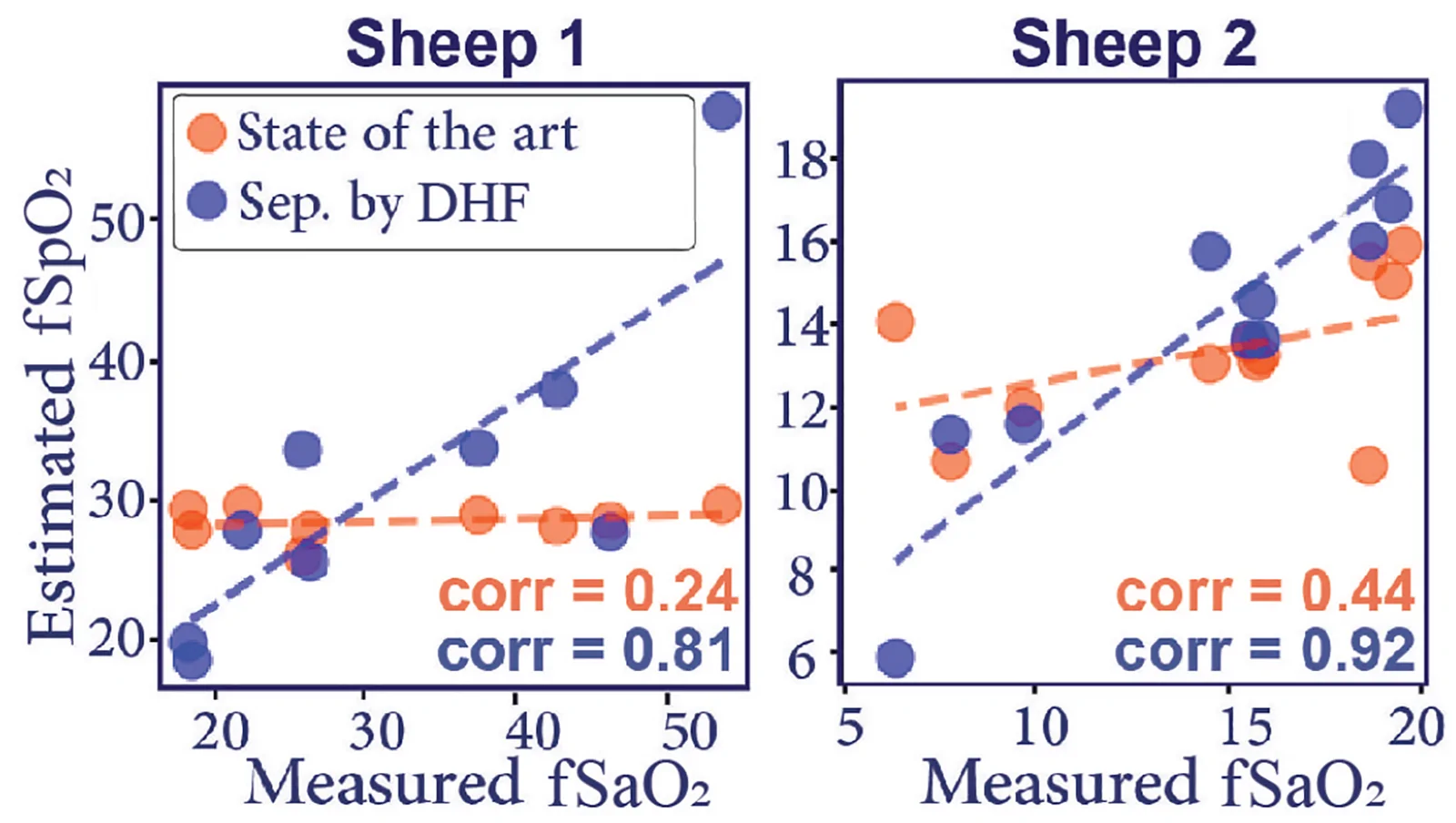
Comparison of fSpO_2_ estimation using fetal signal separated by DHF vs. the state of the art [32] in two different animal experiments.

Figure 13 presents the ${\text{fSpO}}_{2}$ estimation performance for two sheep used in the experiments. The plots compare the relationship between estimated fSpO_2_ vs. reference fSaO_2_, when the separated fetal signal is obtained through spectral masking (similar to [32]) vs. the proposed method, DHF.

Comparing the correlation between fSpO_2_ estimations and fSaO_2_ readings, DHF improves the correlation from 0.24 to 0.81, and from 0.44 to 0.92 in sheep 1, and sheep 2, respectively. The comparison can be stated as an average of 80.5% error improvement relative to the ideal case, i.e., correlation of 1 between fSpO_2_ estimations and fSaO_2_ readings.

## 6 Conclusion

Limitations in dataset quality and quantity, present in many quasi-periodic signal separation applications in wearable systems, have hindered their success despite their significant potential. Incorporation of application-specific prior knowledge can enhance signal separation performance with limited data. In this work, we use the source frequencies (captured through additional sensors or posterior analytical methods) to, first, mask the undesired frequencies for a specific target source, and second, transform the time-frequency representation to create a stronger alignment between the target source and implicit deep priors built into the structure of our proposed neural network. We showcase our method in the TFO application, using both synthesized and *in vivo* data, where it shows significant improvement compared to the state of the art.

Given the importance of quasi-periodic signal separation and the strong dependence of data-limited separation techniques on prior knowledge, further investigation into robust frequency acquisition and estimation strategies is a promising direction for future work. In addition, the network design and preprocessing stages can be further optimized to reduce training latency, by employing strategies such as warm-start initialization or improved background suppression techniques.

## Figures and Tables

**Table 1.** Overview of the Synthesized Mixed Signals

		Syn.	Syn.	Syn.	Syn.	Syn.
		MSig1	MSig2	MSig3	MSig4	MSig5
source1	mean(A)	0.08	0.08	0.4	0.74	0.6
	std(A)	0.02	0.01	0.1	0.1	0.2
	${\text{f}}_{\text{min}}$	0.9	0.8	1.4	0.5	0.5
	${\text{f}}_{\text{max}}$	1.7	1.2	2.3	0.9	0.9
source2	mean(A)	0.03	0.06	0.03	0.08	0.07
	std(A)	0.01	0.02	0.01	0.01	0.01
	${\text{f}}_{\text{min}}$	1.8	1.0	1.6	1.1	1.0
	${\text{f}}_{\text{max}}$	3.0	2.1	3.0	1.8	2.0
source3	mean(A)	–	–	–	0.06	0.04
	std(A)	–	–	–	0.01	0.01
	${\text{f}}_{\text{min}}$	–	–	–	1.8	2.1
	${\text{f}}_{\text{max}}$	–	–	–	2.9	3.5
noise	mean	0.0	0.0	0.0	0.0	0.0
	std	0.003	0.01	0.04	0.01	0.001

**Table 2.** Performance Comparison of Various Signal Separation Methods Applied on Synthesized Mixed Signals Msig1 to MSig5

		EMD [9]		VMD [2]		NMF [14]		REPET [21]		REPET-Ext. [21]		Spect. Masking [6]		DHF	
		SDR(db)	MSE	SDR(db)	MSE	SDR(db)	MSE	SDR(db)	MSE	SDR(db)	MSE	SDR(db)	MSE	SDR(db)	MSE
Syn. MSig1	source1	−1.38	7.4e-4	7.32	1.5e-4	−9.03	8.9e-4	4.68	2.0e-4	9.91	1.0e-4	12.31	6.4e-5	**21.92**	**7.0e-6**
	source2	−6.17	1.3e-4	3.17	1.1e-4	−7.53	1.3e-4	−0.77	6.4e-05	−10.82	1.1e-4	6.44	3.3e-5	**18.30**	**2.6e-6**
Syn. MSig2	source1	−6.36	9.1e-4	3.14	7.1e-4	−4.58	7.8e-4	0.09	4.8e-4	4.82	3.4e-4	4.51	3.5e-4	**12.37**	**5.7e-5**
	source2	−21.75	7.2e-4	−21.06	7.0e-4	−4.98	6.4e-4	−1.25	4.5e-4	−6.2	4.4e-4	1.16	5.6e-4	**11.30**	**5.3e-5**
Syn. MSig3	source1	5.65	5.3e-3	7.24	3.9e-3	−8.79	2.2e-2	6.59	3.3e-3	14.36	8.1e-4	**26.95**	**5.7e-5**	15.81	7.2e-4
	source2	0.07	2.6e-4	−0.15	1.8e-4	−0.18	8.3e-4	−0.04	2.7e-4	−1.63	2.1e-4	−17.3	9.9e-3	**7.79**	**3.0e-5**
Syn. MSig4	source1	5.2	1.1e-2	15.16	1.5e-3	−4.95	3.6e-2	3.83	9.9e-3	18.19	7.8e-4	23.81	2.2e-4	**30.23**	**5.0e-5**
	source2	0.36	9.5e-4	0.76	8.7e-4	−2.63	1.0e-3	−0.11	9.3e-4	−4.29	6.0e-4	4.03	3.8e-4	**13.12**	**4.7e-5**
	source3	−13.79	4.0e-4	−19.95	4.0e-4	−5.59	4.6e-4	−15.76	3.9e-4	−7.26	3.2e-4	8.9	5.3e-5	**14.37**	**1.5e-5**
Syn. MSig5	source1	2.11	1.6e-2	15.53	1.1e-3	−4.31	2.6e-2	1.26	1.1e-2	18.81	5.2e-4	19.26	4.2e-4	**24.69**	**1.3e-4**
	source2	−5.27	7.4e-4	1.02	7.0e-4	−5.64	7.2e-4	−0.05	7.3e-4	−4.42	4.3e-4	1.27	5.5e-4	**18.53**	**1.1e-5**
	source3	−18.59	1.2e-4	3.01	1.1e-4	−10.47	1.2e-4	−11.59	1.2e-4	−7.82	1.0e-4	6.82	2.7e-5	**11.51**	**8.7e-6**
	Average	0.10	9.5e-4	8.69	5.0e-4	−4.84	1.4e-3	1.49	6.7e-4	11.86	3.2e-4	18.56	2.1e-4	**21.71**	**3.0e-5**

Best performance for each source is highlighted in bold.

**Table 3.** Performance Comparison of Competing Signal Separation Methods on Source-Specific Filtered Synthesized Mixed Signals

		EMD [9]		VMD [2]		NMF [14]		REPET [21]		REPET-Ext. [21]		Spect. Masking [6]		DHF	
		SDR(db)	MSE	SDR(db)	MSE	SDR(db)	MSE	SDR(db)	MSE	SDR(db)	MSE	SDR(db)	MSE	SDR(db)	MSE
Syn. MSig1	source1	7.45	1.8e-4	0.85	1.0e-3	−8.98	8.9e-4	5.03	1.6e-4	11.01	6.8e-05	12.31	6.4e-5	**21.92**	**7.0e-6**
	source2	2.21	7.1e-05	1.49	1.4e-4	−8.24	1.2e-4	3.37	3.2e-05	4.83	3.1e-05	6.44	3.3e-5	**18.30**	**2.6e-6**
Syn. MSig2	source1	2.6	4.5e-4	1.83	1.0e-3	−5.3	7.7e-4	1.74	3.1e-4	4.92	2.7e-4	4.51	3.5e-4	**12.37**	**5.7e-5**
	source2	1.17	4.9e-4	1.1	7.5e-4	−5.49	5.7e-4	0.81	3.5e-4	2.58	4.2e-4	1.16	5.6e-4	**11.30**	**5.3e-5**
Syn. MSig3	source1	1.26	9.9e-3	13.7	1.0e-3	−8.73	2.2e-2	6.62	2.9e-3	16.08	5.3e-4	**26.95**	**5.7e-5**	15.81	7.2e-4
	source2	0.04	1.8e-4	0.04	3.1e-4	−0.25	6.9e-4	0.02	2.0e-4	0.06	1.8e-4	−17.3	9.9e-3	**7.79**	**3.0e-5**
Syn. MSig4	source1	4.07	1.4e-2	9.71	6.1e-3	−4.96	3.6e-2	9.54	3.6e-3	18.29	6.7e-4	23.81	2.2e-4	**30.23**	**5.0e-5**
	source2	2.74	4.5e-4	0.27	1.4e-3	−7.06	7.6e-4	2.16	2.8e-4	4.95	2.6e-4	4.03	3.8e-4	**13.12**	**4.7e-5**
	source3	7.19	7.3e-05	−5.3	2.7e-4	−9.11	3.5e-4	4.49	6.5e-05	6.43	5.5e-05	8.9	5.3e-5	**14.37**	**1.5e-5**
Syn. MSig5	source1	0.7	1.9e-2	14.43	1.4e-3	−4.32	2.6e-2	6.26	4.8e-3	18.5	5.0e-4	19.26	4.2e-4	**24.69**	**1.3e-4**
	source2	0.83	4.9e-4	3.53	4.5e-4	−5.2	6.2e-4	1.07	2.9e-4	3.42	3.0e-4	1.27	5.5e-4	**18.35**	**1.1e-5**
	source3	5.64	3.7e-05	0.43	1.2e-4	−8.73	1.1e-4	2.82	2.7e-05	5.06	2.3e-05	6.82	2.7e-5	**11.51**	**8.7e-6**
	Average	3.74	5.5e-4	7.76	6.5e-4	−5.51	1.2e-3	4.59	3.1e-4	12.50	1.7e-4	18.56	2.1e-4	**21.71**	**3.0e-5**

Spectral masks (filters) are applied prior to signal separation methods to retain the signal energy only around the target frequency. Best performance for each source is highlighted in bold.

**Table 4.** DHF Computation Analysis for the Decomposition of Synthetic Mixed Signals

		Mean	Total	Max
		Time (s)	Time (s)	Mem (MB)
Syn. MSig1	source1	25.19	118.75	699.47
	source2	43.20		1454.11
Syn. MSig2	source1	25.76	77.89	731.87
	source2	26.12		775.48
Syn. MSig3	source1	30.05	82.23	902.87
	source2	36.80		1160.15
Syn. MSig4	source1	21.69	121.16	562.07
	source2	30.57		923.53
	source3	40.13		1273.12
Syn. MSig5	source1	17.81	138.27	368.22
	source2	29.65		898.07
	source3	46.29		1517.28

The table reports the mean processing time per source, mean total execution time, and peak memory usage collected from ten repeated runs per data sample for averaging.

## CCS Concepts:

• Computing methodologies → Machine learning; • Applied computing → Life and medical sciences;

## References

[1] Chen Shiqian, Dong Xingjian, Peng Zhike, Zhang Wenming, and Meng Guang. 2017. Nonlinear chirp mode decomposition: A variational method. IEEE Transactions on Signal Processing 65, 22 (2017), 6024–6037.

[2] Dragomiretskiy Konstantin and Zosso Dominique. 2013. Variational mode decomposition. IEEE Transactions on Signal Processing 62, 3 (2013), 531–544.

[3] Eriksen Thomas and Rehman Naveed ur. 2023. Data-driven nonstationary signal decomposition approaches: A comparative analysis. Scientific Reports 13, 1 (2023), 1798. 10.1038/s41598-023-28390-w PMC9889333 36721010

[4] Fong Daniel D., Yamashiro Kaeli J., Vali Kourosh, Galganski Laura A., Thies Jameson, Moeinzadeh Rasta, Pivetti Christopher, Knoesen André, Srinivasan Vivek J., Hedriana Herman L., 2020. Design and in vivo evaluation of a non-invasive transabdominal fetal pulse oximeter. IEEE Transactions on Biomedical Engineering 68, 1 (2020), 256–266. 10.1109/TBME.2020.3000977 32746021

[5] Freeman Roger K.. 2002. Problems with intrapartum fetal heart rate monitoring interpretation and patient management. Obstetrics & Gynecology 100, 4 (2002), 813–826. DOI: 10.1016/s0029-7844(02)00343-1 12383554

[6] Gerkmann Timo and Vincent Emmanuel. 2018. Spectral masking and filtering. Audio Source Separation and Speech Enhancement. John Wiley & Sons, 65–85.

[7] Goldberger Ary L., Amaral Luis AN, Glass Leon, Hausdorff Jeffrey M., Ivanov Plamen Ch, Mark Roger G., Mietus Joseph E., Moody George B., Peng Chung-Kang, and Stanley H. Eugene. 2000. PhysioBank, PhysioToolkit, and PhysioNet: Components of a new research resource for complex physiologic signals. Circulation 101, 23 (2000), e215–e220. 10.1161/01.cir.101.23.e215 10851218

[8] Harmouche Jinane, Fourer Dominique, Auger François, Borgnat Pierre, and Flandrin Patrick. 2017. The sliding singular spectrum analysis: A data-driven nonstationary signal decomposition tool. IEEE Transactions on Signal Processing 66, 1 (2017), 251–263.

[9] Huang Norden E., Shen Zheng, Long Steven R., Wu Manli C., Shih Hsing H., Zheng Quanan, Yen Nai-Chyuan, Tung Chi Chao, and Liu Henry H.. 1998. The empirical mode decomposition and the Hilbert spectrum for nonlinear and non-stationary time series analysis. Proceedings of the Royal Society of London. Series A: Mathematical, Physical and Engineering Sciences 454, 1971 (1998), 903–995.

[10] Isik Yusuf, Le Roux Jonathan, Chen Zhuo, Watanabe Shinji, and Hershey John R.. 2016. Single-channel multi-speaker separation using deep clustering. arXiv:1607.02173. Retrieved from https://arxiv.org/abs/1607.02173

[11] Jansson Andreas, Humphrey Eric, Montecchio Nicola, Bittner Rachel, Kumar Aparna, and Weyde Tillman. 2017. Singing voice separation with deep u-net convolutional networks. In 18th International Society for Music Information Retrieval Conference. (2017).

[12] Johnson Alistair, Bulgarelli Lucas, Pollard Tom, Horng Steven, Celi Leo Anthony, and Mark Roger. 2020. Mimic-iv. PhysioNet. Retrieved August 23, 2021 from https://physionet.org/content/mimiciv/1.0/

[13] Kasap Begum, Vali Kourosh, Qian Weitai, Saffarpour Mahya, and Ghiasi Soheil. 2023. KUBAI: Sensor fusion for non-invasive fetal heart rate tracking. IEEE Transactions on Biomedical Engineering 70, 7 (2023), 2193–2202. DOI: 10.1109/TBME.2023.3238736 PMC10346940 37022063

[14] Lee Daniel D. and Seung H. Sebastian. 1999. Learning the parts of objects by non-negative matrix factorization. Nature 401, 6755 (1999), 788–791. 10.1038/44565 10548103

[15] Luo Yi, Chen Zhuo, Hershey John R., Le Roux Jonathan, and Mesgarani Nima. 2017. Deep clustering and conventional networks for music separation: Stronger together. In Proceedings of the 2017 IEEE International Conference on Acoustics, Speech and Signal Processing (ICASSP). IEEE, 61–65. 10.1109/ICASSP.2017.7952118 PMC5791533 29398973

[16] Luo Yi and Mesgarani Nima. 2018. Tasnet: Time-domain audio separation network for real-time, single-channel speech separation. In Proceedings of the 2018 IEEE International Conference on Acoustics, Speech and Signal Processing (ICASSP). IEEE, 696–700.

[17] Luo Yi and Mesgarani Nima. 2019. Conv-tasnet: Surpassing ideal time–frequency magnitude masking for speech separation. IEEE/ACM Transactions on Audio, Speech, and Language Processing 27, 8 (2019), 1256–1266. 10.1109/TASLP.2019.2915167 PMC6726126 31485462

[18] Narayanaswamy Vivek, Thiagarajan Jayaraman J., Anirudh Rushil, and Spanias Andreas. 2020. Unsupervised audio source separation using generative priors. arXiv:2005.13769. Retrieved from https://arxiv.org/abs/2005.13769

[19] Nelson Karin B., Sartwelle Thomas P., and Rouse Dwight J.. 2016. Electronic fetal monitoring, cerebral palsy, and caesarean section: Assumptions versus evidence. BMJ 355 (2016). 10.1136/bmj.i6405 PMC6883481 27908902

[20] Niedźwiecki Maciej and Kaczmarek Piotr. 2005. Estimation and tracking of complex-valued quasi-periodically varying systems. Automatica 41, 9 (2005), 1503–1516.

[21] Rafii Zafar and Pardo Bryan. 2012. Repeating pattern extraction technique (REPET): A simple method for music/voice separation. IEEE Transactions on Audio, Speech, and Language Processing 21, 1 (2012), 73–84.

[22] Rixen Joel and Renz Matthias. 2022. Sfsrnet: Super-resolution for single-channel audio source separation. In Proceedings of the AAAI Conference on Artificial Intelligence 36 (2022), 11220–11228.

[23] Ronneberger Olaf, Fischer Philipp, and Brox Thomas. 2015. U-net: Convolutional networks for biomedical image segmentation. In Medical Image Computing and Computer-Assisted Intervention–MICCAI 2015: 18th International Conference, Proceedings, Part III 18. Springer, 234–241.

[24] Saffarpour Mahya, Vali Kourosh, Qian Weitai, Kasap Begum, Farmer Diana L., Wang Aijun, and Ghiasi Soheil. 2024. Deep harmonic finesse: Signal separation in wearable systems with limited data. In Proceedings of the 2024 61st ACM/IEEE Design Automation Conference (DAC). IEEE.

[25] Saffarpour Mahya, Vali Kourosh, Qian Weitai, Kasap Begum, Farmer Diana L., Wang Aijun, and Ghiasi Soheil. 2024. Deep quasi-periodic priors: Signal separation in wearable systems with limited data. In Proceedings of the 2024 Design, Automation & Test in Europe Conference & Exhibition (DATE). IEEE.

[26] Sinex JE. 1999. Pulse oximetry: Principles and limitations. The American Journal of Emergency Medicine 17, 1 (1999), 59–67. DOI: 10.1016/s0735-6757(99)90019-0 9928703

[27] Smith Aaron Asael, Li Rui, and Tse Zion Tsz Ho. 2023. Reshaping healthcare with wearable biosensors. Scientific Reports 13, 1 (2023), 4998. 10.1038/s41598-022-26951-z PMC10043012 36973262

[28] Stoller Daniel, Ewert Sebastian, and Dixon Simon. 2018. Wave-u-net: A multi-scale neural network for end-to-end audio source separation. In IEEE International Conference on Acoustics, Speech, and Signal Processing (ICASSP).

[29] Tian Yapeng, Xu Chenliang, and Li Dingzeyu. 2020. Deep audio prior: Learning sound source separation from a single audio mixture. In Proceedings of the IEEE/CVF Conference on Computer Vision and Pattern Recognition Workshops.

[30] Tzinis Efthymios, Wisdom Scott, Jansen Aren, Hershey Shawn, Remez Tal, Ellis Daniel PW, and Hershey John R.. 2020. Into the wild with audioscope: Unsupervised audio-visual separation of on-screen sounds. In International Conference on Learning Representations.

[31] Ulyanov Dmitry, Vedaldi Andrea, and Lempitsky Victor. 2018. Deep image prior. In Proceedings of the IEEE Conference on Computer Vision and Pattern Recognition. 9446–9454.

[32] Vali Kourosh, Kasap Begum, Qian Weitai, Vafi Ata, Saffarpour Mahya, and Ghiasi Soheil. 2021. Estimation of fetal blood oxygen saturation from transabdominally acquired photoplethysmogram waveforms. In Proceedings of the 2021 43rd Annual International Conference of the IEEE Engineering in Medicine & Biology Society (EMBC). IEEE, 1100–1103. 10.1109/EMBC46164.2021.9629515 34891479

[33] Wisdom Scott, Tzinis Efthymios, Erdogan Hakan, Weiss Ron, Wilson Kevin, and Hershey John. 2020. Unsupervised sound separation using mixture invariant training. Advances in Neural Information Processing Systems 33 (2020), 3846–3857.

[34] Zhang Geliang and Godsill Simon. 2016. Fundamental frequency estimation in speech signals with variable rate particle filters. IEEE/ACM Transactions on Audio, Speech, and Language Processing 24, 5 (2016), 890–900.

[35] Zhang Zhoutong, Wang Yunyun, Gan Chuang, Wu Jiajun, Tenenbaum Joshua B., Torralba Antonio, and Freeman William T.. 2019. Deep audio priors emerge from harmonic convolutional networks. In Proceedings of the International Conference on Learning Representations.

